# Patients report more severe daily limitations than recognized by their physicians

**DOI:** 10.1002/clc.23269

**Published:** 2019-09-30

**Authors:** Renata R. T. Castro, Emer Joyce, Neal K. Lakdawala, Garrick Stewart, Anju Nohria, Michael M. Givertz, Akshay Desai, Eldrin F. Lewis, Lynne W. Stevenson

**Affiliations:** ^1^ Cardiovascular Division, Department of Medicine Brigham and Women's Hospital Boston Massachusetts; ^2^ Hospital Naval Marcilio Dias Rio de Janeiro Brazil; ^3^ School of Medicine, Universidade Iguaçu Rio de Janeiro Brazil; ^4^ Department of Cardiovascular Medicine Cleveland Clinic Foundation Cleveland Ohio; ^5^ Cardiomyopathy & Advanced Heart Disease Training Vanderbilt Heart and Vascular Institute Nashville Tennessee

**Keywords:** cardiomyopathy, cardiovascular disease, heart failure

## Abstract

**Background:**

Patient limitations guide selection of heart failure therapies, for which indications often specify New York Heart Association Class.

**Objectives:**

To determine the extent of patient‐reported limitations during daily activities and compare to New York Heart Association class assigned by providers during the same visit, and to left ventricular ejection fraction (LVEF) group.

**Methods and Results:**

While waiting for their appointment, 948 patients on return visits to an ambulatory HF clinic completed a written questionnaire assessing specific activity limitations, which were compared to physician‐assigned NYHA class during the same visit. Patient‐reported limitation to perform daily activity ranged from 25% for bathing to 61% for yardwork or housework and 71% for jogging or hurrying. Most patients who did not report limitations to perform daily life activities were correctly classified as NYHA I by the physicians (76%), but 12% of the 376 patients classified as NYHA I reported limitations to showering or bathing and 73% reported limitations while doing yardwork or house work. Limitation to walking was reported by 172 patients (50%) classified as class II. Limitations to walking one block were most common in patients with LVEF ≥40% compared to patients with LVEF <40%, and least commonly, in HF with better EF (improved from 31 ± 13 to 52 ± 7).

**Conclusions:**

Activity limitations are commonly reported by ambulatory HF patients, but underestimated by physicians. It is not clear how this should guide therapy validated for NYHA class but focused activity questions may merit wider use to track limitations and improvement in ambulatory HF.

## 1 INTRODUCTION

Increasing penetration of recommended therapies for heart failure (HF) has improved outcomes,^1^ but morbidity and mortality remain substantial.^2^, ^3^ Heart failure guidelines mandate consideration not only for prolonging survival but also on improving quality of life (QOL),^4^, ^5^ upon which patients may place similar or greater value.^6^

A major contributor to QOL in HF is functional capacity, for which impairment can lead to loss of independence and reduced self‐esteem.^7^, ^8^ The New York Heart Association (NYHA) functional classification system^9^ is widely used in clinical practice, in clinical trials,^10^ in translation of trials into guideline recommendations for therapies,^11^ and in decisions about advanced HF therapies.^12^, ^13^ Understanding functional status and its improvement may be further enhanced by consideration of patient reported limitations to specific activities during daily life, as assessed by some of the questions in the Kansas City Cardiomyopathy Questionnaire.^14^, ^15^ Although NYHA classification has been revised several times since 1928,^9^ it still relies on physicians' subjective estimate of a patient's ability to carry out “ordinary” activity. Consequently, the decision of a physician to classify a patient per NYHA functional class will depend on both patient and physician interpretation of “ordinary physical activity” and how to grade limitations as slight or marked.^16^, ^17^ Although NYHA class remains a robust discriminator of clinical outcome, reproducibility is low when the same patients are evaluated by different physicians.^14^, ^17^, ^18^ In order to focus on patient‐centered outcomes,^4^ it would be potentially useful both in heart failure clinics and in clinical trials to consider patient's individual perceptions about limitations, adding information to frequently used NYHA classification. Although the nature of heart failure symptoms may be similar across ejection fraction (EF) groups, there are differences in demographics and comorbidities, such that the nature and degree of limitations may vary.^19^

We hypothesized that patients may report limitations to specific daily activity that is not well‐reflected in physician‐assigned NYHA classification. The major aim of the present study was to determine the extent of patient‐reported limitations during daily activities and to compare with the physician assignment of NYHA class on the same day. A further aim was to compare the frequency of perceived limitations across the HF different left ventricular ejection fraction (LVEF) types.

## 2 METHODS

### 2.1 Patient population and protocol

The present study is an observational cross‐sectional analysis of patients seen in the ambulatory HF clinic at Brigham and Women's Hospital. As part of a quality improvement initiative to improve communication about quality of life, patients were asked to complete a questionnaire, while waiting for routine clinical visit. The questionnaire was handed to the clinic physician beneath the standard registration/vital sign form as the visit began. The clinic visit and the associated clinical note were completed directly by attending physicians, who had finished formal training in heart failure. There were no specific instructions regarding how to review or incorporate the information in the clinical assessment or documentation.

Patients who did not provide information about limitations to perform daily life activities, those with unknown LVEF and those whose physicians did not assign a NYHA class in the chart were excluded from the present analysis.

### 2.2 Patient questionnaire

The two‐sided, single page, self‐administered questionnaire included questions about limitations to perform daily life activities, quality of life, symptoms, and patients' perception about clinical stability. To assess self‐perceived limitation to perform daily life activities, the questionnaire included the following question: “Please indicate how much you are limited by your heart failure (shortness of breath or fatigue) in your ability to do the following over the last 4 weeks?”. The activities listed included showering/bathing; walking one city block on level ground; yardwork, housework, or carrying groceries; climbing a flight of stairs (10 steps) without stopping; and hurrying or jogging (such as to catch a bus).^15^ The degree of each limitation was classified as not at all limited; slightly limited; moderately limited; quite a bit limited; or extremely limited. Additional questions related to recent hospitalizations, ICD shocks, orthopnea, and overall quality of life.

### 2.3 Clinical data

Patient demographics and clinical characteristics, including HF etiology and medical comorbidities, were assessed from clinical charts. NYHA functional class and current medications on the day of the clinic visit were updated in the electronic medical record by the attending physician, as part of routine assessment and included in our database.

LVEF was recorded from the most recent available echocardiogram within 18 months from the clinical visit. HF with reduced ejection fraction (HFrEF) was defined as LVEF <40%. HF with preserved ejection fraction (HFpEF) was identified by the clinical history of HF with left ventricle ejection fraction (LVEF) ≥40% without any previous echocardiogram with LVEF <40%. HF with better EF (HFbEF) was defined when prior echocardiographic EF had been <40% with subsequent improvement of at least 10% of LVEF in the present echocardiogram.^20^, ^21^

The Institutional Review Board of Brigham and Women's Hospital approved this retrospective observational study. No consents were obtained for the questionnaires, which were administered to patients as part of a clinical care and quality improvement initiative.

### 2.4 Statistical analysis

Initially, normality of distribution was tested, validating the use of parametric statistics. Continuous variables are presented as mean ± SD and categorical variables are presented as frequencies and percentages. Multiple‐group comparisons were performed using analysis of variance or chi‐square tests as appropriate. Post‐hoc correction for multiple comparisons between groups was performed using the Bonferroni method.

To compare patient‐perceived daily life activity limitations across LVEF‐HF types (HFrEF, HFpEF, and HFbEF), we performed multivariate logistic regression adjusted by age, body mass index, and comorbidities.

All statistical tests were two‐sided, and *P* values <.05 were considered to indicate statistical significance. Statistical analysis was performed using Stata version 14 (Stata Corp, College Station, TX).

## 3 RESULTS

**Figure 1:**
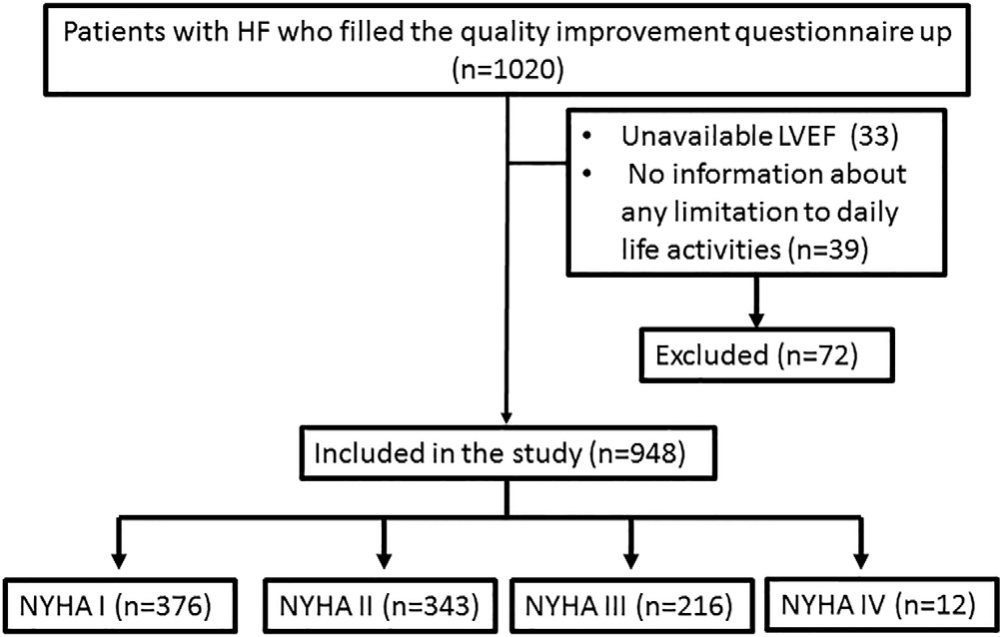
Patient's flow diagram. HF, heart failure; LVEF, left ventricular ejection fraction; NYHA, New York Heart Association functional classification

A total of 1020 patients with HF completed a questionnaire on the day of their routine office visit. Patients were excluded for lack of recent measurement of LVEF (n = 33) or incomplete information about limitations to perform daily life activities (n = 39). The following analysis included the remaining 948 patients. The majority of patients were classified by physicians as NYHA I (n = 376, 40%); 343 were NYHA II (36%), 216 were NYHA III (23%), and 12 were NYHA IV (1%) (Figure 1).

Patients classified as NYHA I by the HF physicians were younger, had lower body mass index, and presented less comorbidities as diabetes and chronic kidney disease than the ones classified as NYHA II or III (Table 1). The majority of patients (75%) reported limitations to daily life activities, most commonly to hurrying or catching a bus (71%). Limitation to walking a block was reported by 45%, and 25% perceived some limitation even to bathing.

**Table 1:** Patients demographics and clinical characteristics accordingly to NYHA functional classification

	All patients	NYHA I	NYHA II	NYHA III	NYHA IV	*P* value between NYHA (I‐III) groups
Number of patients (%)	948 (100.0)	376 (40)	343 (36)	216 (23)	12 (1)	
*Demographics*						
Age (years)	57 ± 16	52 ± 16*	58 ± 15*	61 ± 15*	62 ± 10	<.001
BMI (kg/m^2^)	30 ± 7	29 ± 6*	30 ± 6*	31 ± 8*	30 ± 11	<.001
Female, n (%)	379 (40)	141 (37)	143 (42)	90 (42)	5 (42)	.65
LVEF (%)	42 ± 16	46 ± 15*	40 ± 16**	39 ± 18**	39 ± 17	<.001
*Comorbidities*						
Diabetes mellitus (%)	210 (22)	48 (13)*	89 (26)**	66 (31)**	7 (58)	<.001
Obstructive lung disease (%)	149 (16)	32 (8)*	60 (17)**	50 (23)**	7 (58)	<.001
Chronic kidney disease (%)	133 (14)	25 (7)*	58 (17)**	45 (21)**	5 (42)	<.001
ICM (%)	156 (16)	34 (9)*	79 (23)**	41 (19)**	1 (8)	<.001
Hypertension (%)	432 (46)	139 (37)*	160 (47)*	124 (57)*	8 (67)	.003
Atrial fibrillation/flutter (%)	260 (26)	65 (17)*	100 (29)**	79 (37)**	5 (42)	<.001
*Therapies*						
ICD (%)	244 (39)	77 (31)*	105 (44)**	59 (44)**	2 (20)	.012
CRTD (%)	95 (20)	17 (10)*	41 (22)**	34 (31)**	3 (30)	<.001
ACE inhibitors/ARBs (%)	678 (72)	263 (70)	253 (74)	153 (71)	9 (75)	.70
Beta‐blockers (%)	753 (79)	276 (73)*	289 (84)**	177 (82)**	11 (92)	.002
Diuretic agents (%)	544 (57)	141 (37)*	220 (64)*	172 (80)*	11 (92)	<.001
Digoxin (%)	216 (23)	56 (15)*	80 (23)*	75 (35)*	5 (42)	<.001
Warfarin (%)	307 (32)	85 (23)*	122 (36)**	93 (43)*	7 (58)	<.001
Amiodarone (%)	102 (11)	29 (8)***	48 (14)**	22 (10)	3 (25)	.019

Abbreviations: ACE, angiotensin converting enzyme; ARB, angiotensin receptor blocker; BMI, body mass index; CRT, cardiac resynchronization therapy; LVEF, left ventricle ejection fraction; ICD, implantable cardioverter defibrillator; ICM, ischemic cardiomyopathy.

*P < .05 vs all other groups; **P < .05 vs NYHA I; ***P < .05 vs NYHA II.

**Figure 2:**
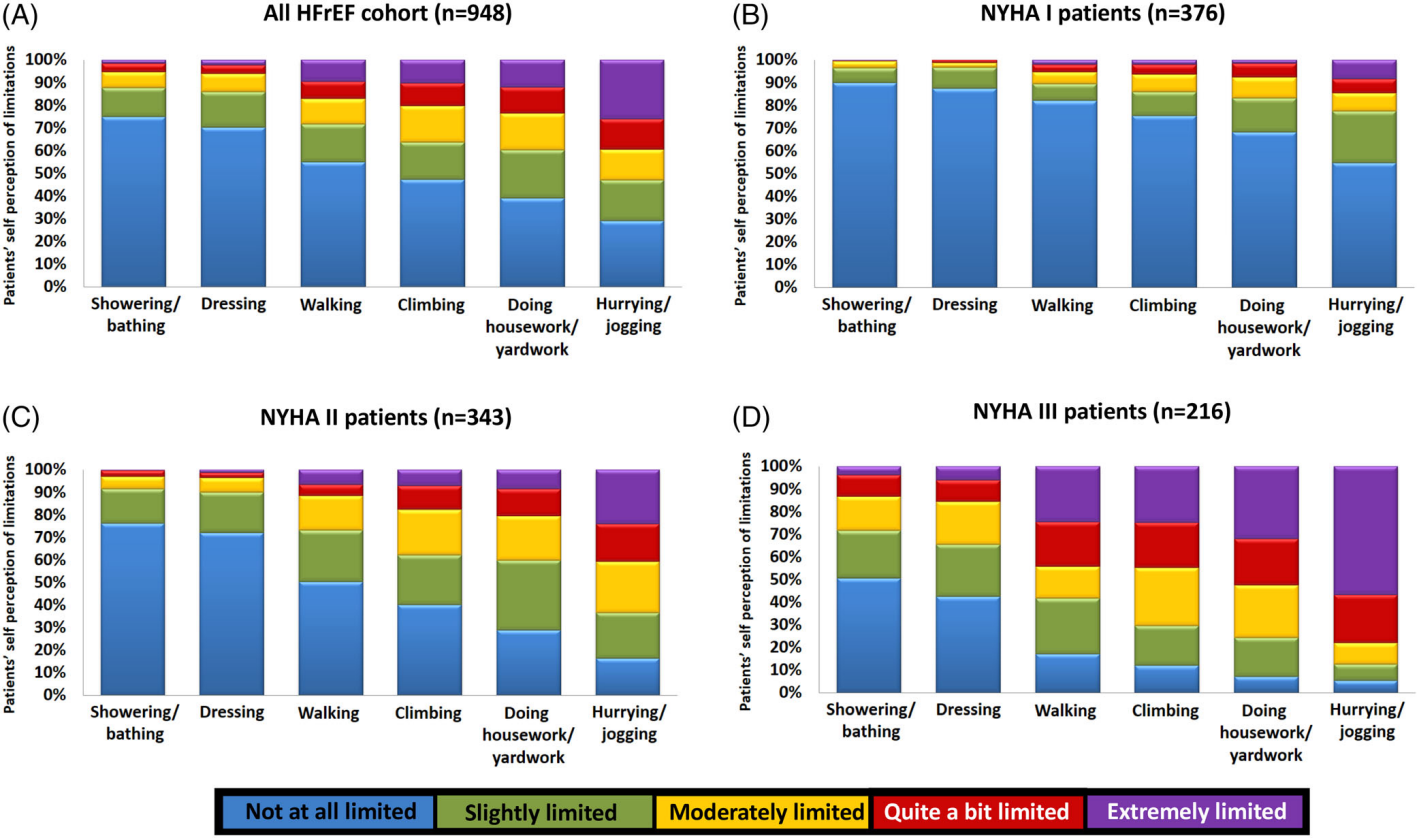
Degree of limitations to perform daily activities reported by ambulatory heart failure patients. Panel A includes all patients (NYHA I to IV; n = 948) and panels B, C, and D include patients designated as NYHA class I (n = 376), II (n = 343), and III (n = 216), respectively. Colors indicate the perceived degree of limitation for each activity

While the frequency and degree of limitations were reported as higher by patients with higher NYHA designation (Figure 2), under‐classification of NYHA by physicians was common.

Almost 12% of patients classified as NYHA I by HF physicians reported limitations to showering and bathing, and 20% of them presented limitations to walk one block on level ground. Among patients designated as class II, 52% described limitation, while walking a block and 73% perceived limitations, while doing housework or yardwork.

**Figure 3:**
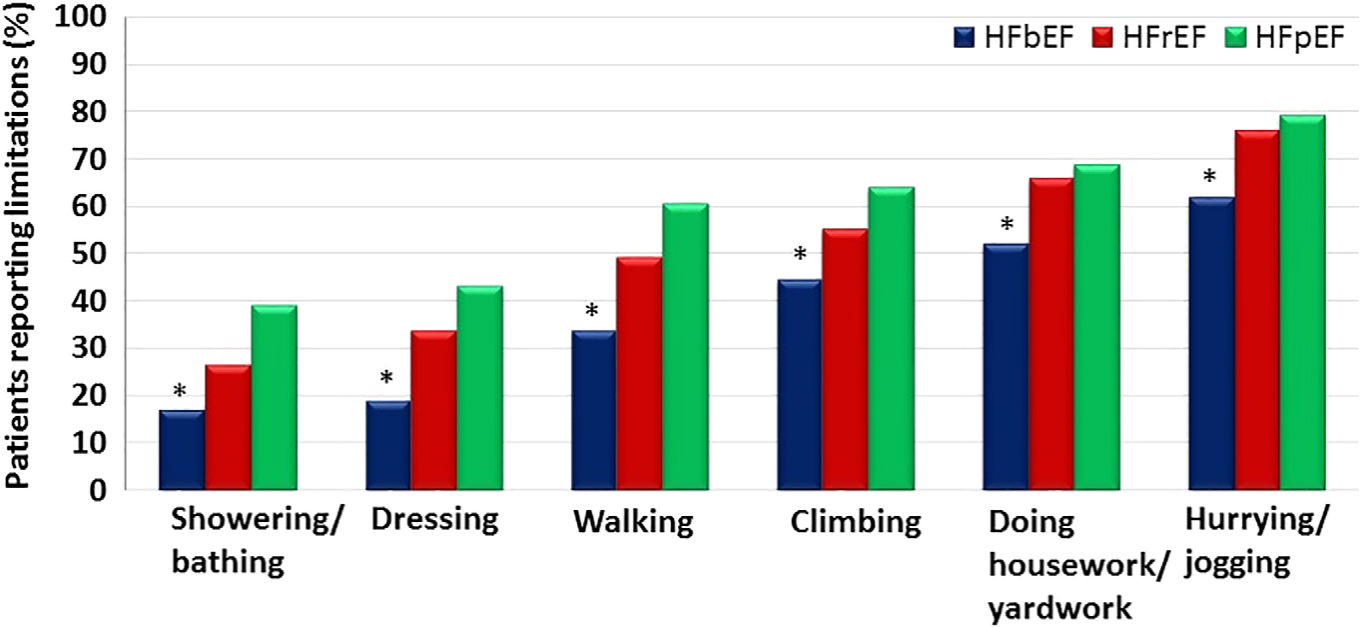
Percentage of patients reporting limitations to perform daily life activities accordingly to left ventricular ejection fraction subtype. *P < .01 vs HFpEF & HFrEF. *P* values from multivariate logistic regression adjusted by age, body mass index, and comorbidities. HFrEF, heart failure with reduced ejection fraction (n = 400); HFbetterEF, heart failure with better ejection fraction (n = 382); HFpEF, heart failure with preserved ejection fraction (n = 166)

When patients were distributed accordingly to LVEF group (Table 2), most of them were classified as HFrEF (n = 400, LVEF: 26 ± 7%), followed by HFbetterEF EF (n = 382, LVEF: 52 ± 7%) and HFpEF (n = 166, LVEF: 60 ± 7%). Patients with HFbetterEF reported the fewest limitations to daily life activities after adjusting for age, gender, and comorbidities (Figure 3). Although patients with HFpEF presented higher frequency of limitations when compared to patients with HFrEF in the univariate analysis, the significance of this comparison disappeared in the regression model adjusted for demographic characteristics and comorbidities.

**Table 2:** Patients demographics and comorbidities accordingly to left ventricular ejection fraction subtype

	HFrEF	HFbEF	HFpEF	*P* value between LVEF groups
Number of patients (%)	400 (42)	382 (40)	166 (18)	
*Demographics*				
Age (years)	52 ± 14*	54 ± 16*	64 ± 16*	<.001
BMI (kg/m^2^)	29 ± 7	30 ± 7	30 ± 8	.34
Female, n (%)	111 (28)	175 (46)	93 (56)	.65
LVEF (%)	26 ± 7*	52 ± 8*	60 ± 7*	<.001
*Comorbidities*				
Diabetes mellitus (%)	100 (25)	58 (15) ** ***	52 (31)	<.001
Obstructive lung disease (%)	57 (14)	57 (15)	35 (21)	.11
Chronic kidney disease (%)	67 (17)	33 (9)** ***	33 (20)	<.001
ICM (%)	102 (25)*	54 (14)*	0 (00)*	<.001
Hypertension (%)	166 (42)***	163 (43)***	103 (62)**	<.001
History of atrial fibrillation/flutter (%)	112 (28)	85 (22) ***	53 (32)	.038

Abbreviations: BMI: body mass index; ICM: ischemic cardiomyopathy; HFbEF: Heart failure with better ejection fraction; HFpEF: Heart failure with reduced ejection fraction; HFrEF: Heart failure with reduced ejection fraction; LVEF: left ventricle ejection fraction.

*P < .05 vs all other groups; **P < .05 vs HFrEF; ***P < .05 vs HFpEF.

## 4 DISCUSSION

Ambulatory patients seen for routine visits in HF clinic reported substantial limitations to daily activities that were often not reflected in the NYHA class as assessed by experienced heart failure clinicians. Patients with HFpEF and HFrEF were more limited in their activities than patients whose previously low‐ejection fraction had improved (HFbetterEF).

### 4.1 Symptom burden in patients with heart failure

The penetrance of guideline‐recommended therapies was high in the present cohort, although the timing of the study preceded the introduction of newer therapies that may further improve functional classification.^11^, ^22^, ^23^, ^24^ The burden of activity limitation was higher than expected for an ambulatory middle‐aged HF population, with more than half of patients reporting limitations to climbing one flight of stairs, 30% of patients reporting limitations to dressing without stopping, and 25% presenting limitations to bathing. Skalska et al^25^ reported a high dependence on assistance for bathing (28%) in a HF cohort, but their patients were much older (mean age 80 years) than our population with mean age of 57 years.

### 4.2 Disparity between patient‐reported limitations and assigned NYHA class

NYHA classification has been widely used in clinical trials as both an enrollment criterion and as an outcome measurement. It has consistently provided reliable discrimination for risk of hospitalizations and death.^14^ Our study confirms that higher NYHA classification discriminates between patients with greater vs fewer limitations to routine activities. However, NYHA classification was not well calibrated to patient‐reported activity limitations, as 10% patients classified as NYHA I described limitations performing low‐intensity activities as showering and dressing, while most class II patients reported limitations when climbing one flight of stairs and during housework or yardwork. Alternatively, 5% of patients classified as NYHA III perceived no limitation to hurrying and jogging, which are moderate to high‐intensity activities. As patients tend to avoid activities that are difficult to perform,^25^ their general perception of ability and their reports to physicians may underestimate their actual limitation for specified activities.

Discrepancies in classifying patients as NYHA II or III has been previously described, not only for differing perceptions between physicians regarding the same patient,^14^ but also by comparisons of NYHA classification with a more objective measurement as “how far a patient can walk”.^16^ While quantitative classification has been established by Weber et al^26^ for clinical class and peak oxygen consumption, there is currently no standard approach of questioning to be consistently employed by physicians to calibrate the NYHA classification with specific activities.

It is not known how the accelerating time demands of ambulatory follow‐up clinics have affected the disparity between patient and physician perception of limitation. After the in‐depth evaluation as a new patient, returning patients may have decreased their activity gradually to match their disease progression, failing to appreciate the cumulative degree of limitation. However, there is likely also an unacknowledged patient‐provider collusion to focus on positive information. The patients may be reluctant to “disappoint” their physician but also resistant to negative news, new prescriptions, or new proscriptions. On their side of the collusion, physicians may consciously or unconsciously communicate their pleasure at favorable information and their time conflict when encountering new issues that warrant additional attention during a busy clinic where other patients are waiting.

### 4.3 Perceived limitations and LVEF subtype

In our study, patients with HFpEF and HFrEF perceived similar limitations to perform daily life activities after adjusting for age, gender, and comorbidities. Depending on the details of the populations studied, QOL impairment has been shown to be similar or slightly different between HFrEF and HFpEF groups.^19^, ^27^ This may reflect the characteristic pathophysiology of each of these LVEF HF subtypes,^28^, ^29^, ^30^, ^31^, ^32^, ^33^, ^34^, ^35^ and the fact that HFpEF in general encompasses older patients in whom age and accumulating comorbidities play an increasing role in limitation.^36^ In our study, the difference between limitations reported by patients with HFrEF and HFpEF disappeared when the regression model was adjusted by age, body mass index, and comorbidities.

More recently, HFbetterEF has been described, beginning with low LVEF and improving to LVEF >0.40 or 0.50, depending on the study.^21^, ^37^ The associated perception of better quality of life and less exercise limitation may reflect better circulatory reserve and also relative framing where they compare their current state to previously more severe limitations. Although patients with HFbetterEF have better survival^38^ and biochemical profile than patients with HFpEF and HFrEF, persistent abnormalities in the neurohormonal profile indicate that HFbEF is rarely truly “recovered.”^21^ Our study expanded these findings, showing that patients with HFbEF still present substantial limitations to daily life activities, as almost 20% of them reported limitations to dressing and the majority reported inability to jogging or hurrying as to catch a bus.

### 4.4 Study limitations

The present study analyzed patient self‐perceived limitations to daily life activity. Factors of resilience, spiritualism, duration of disease, and previous experience and expectations of disease progression^39^ could have influenced these perceptions.^40^, ^41^ These variables were not addressed, and we cannot ascertain if they could have impacted patient perceptions of limitations or discussions with their doctors who assigned the NYHA designation.

Patients filled the questionnaires while waiting for their office visits. Attending physicians had access to the answered questionnaires, which were expected to influence their decisions when classifying NYHA in patient notes. There was no report about how frequently physicians had read or used the information provided by the questionnaires or whether this information influenced their clinical assessment. However, considering that the activity information was provided in real time to the physicians on the same day, the discrepancies between their assessments and the patient descriptions are even more notable.

Results of the present study arise from a HF referral center, where all the physicians providing assessment and care are HF specialists with access to heart transplants and implants of ventricular assist devices when needed. These results may not be generalizable to other care settings, particularly to general medicine clinics where multiple different diseases are assessed simultaneously.

## 5 CONCLUSIONS

The discrepancy between patient perception of activity limitation and the physician estimate of NYHA class suggests that routine outpatient evaluation should incorporate either patient reporting via a separate interface or physician elicitation of specific activity performance as suggested by Albert et al^42^ Discussing the specific activities most important to patients would clearly advance the agenda to render more patient‐centered care.

It is less clear whether reclassification of functional level according to specific activities should refine the selection of current therapies according to the NYHA class designated in the trials that provided the evidence for approval, reimbursement, and guideline recommendations. New trials designed to demonstrate improvement in functional capacity may need to isolate the questions about specific activities from the integrated summary of questionnaires such as the Kansas City Cardiomyopathy Questionnaire^4^ or use the Duke activity status index^43^ within which these activity questions are currently embedded. It is possible that such refinement would enhance recognition of symptomatic benefit or impairment for daily activities during adjustment of therapies. As the choice and titration of therapies become increasingly complex, more precise elicitation and tracking of activity limitations may help guide future interventions indicated to improve functional capacity and quality of life.

## CONFLICT OF INTEREST

The authors declare no potential conflict of interests.
